# Case Report: A four-stage diagnostic cascade: from coronary stents to pancreatic cancer in a patient with recurrent chest pain

**DOI:** 10.3389/fonc.2026.1785461

**Published:** 2026-06-29

**Authors:** Tao Lin, Ziyi Huang, Xiahui Huang

**Affiliations:** 1 Department of Emergency, Quzhou People’s Hospital (Affiliated Quzhou Hospital of Wenzhou Medical University), Quzhou, Zhejiang, China; 2 Department of Pancreatic Disease, Quzhou People’s Hospital (Affiliated Quzhou Hospital of Wenzhou Medical University), Quzhou, Zhejiang, China

**Keywords:** acute pancreatitis, coronary heart disease, misdiagnose, pancreatic uncinate process carcinoma, recurrent chest pain

## Abstract

Chest pain sends millions to emergency rooms, yet its source can be surprisingly elusive. While the focus often falls on the heart, the abdomen can produce pain that perfectly mimics angina, leading clinicians down a deceptive path. This diagnostic trap becomes even more dangerous when a patient already has known coronary artery disease, as familiar symptoms may mask a completely different threat. We present a case that unfolded in an unexpected sequence. A patient with recurrent chest pain underwent coronary stenting, only to have the same pain return soon after. What followed was a clinical puzzle that gradually uncovered not one, but two additional diagnoses—each one more surprising than the last, and each demanding a fundamental shift in thinking. The journey from the catheterization laboratory to the final answer holds sobering lessons about cognitive anchoring, the value of overlooked laboratory clues, and the need to look beyond the heart when chest pain refuses to resolve.

## Introduction

Cardiovascular diseases (CVDs) are the leading cause of death worldwide, with an estimated 17.9 million deaths per year. Acute coronary syndrome (ACS) is a major contributor to CVD mortality and frequently manifests as chest pain (1).,studies indicate that 15-30% of patients undergoing coronary angiography for chest pain have normal results (2),and a significant portion of these “non-cardiac causes” are overlooked in acute care settings (3).Among these non-cardiac origins, intra-abdominal pathologies constitute an important yet frequently underestimated category (4).

Pancreatic cancer (PC) is the third leading cause of cancer death in the United States and the eleventh most common cancer worldwide, with a 5-year survival rate of only 13%. Its incidence, estimated at 0.6% to 1% per year, is expected to make it the second leading cause of cancer death by 2030 (5). Equally important is that acute pancreatitis(AP)—with a global incidence of up to 456 cases per million and an alarming 2.6% annual rise—may serve as the sentinel event of an underlying malignancy (6, 7).

The pain caused by coronary heart disease, acute pancreatitis and pancreatic cancer usually has typical differences (**Table 1**) (8, 9), but inflammatory or neoplastic pancreatic diseases can mimic cardiac chest pain by irritating the phrenic nerve sometimes, the retroperitoneal nerve plexus, or by causing referred pain, often leading to initial misdiagnosis (7, 10, 11). The value of this case lies in its first complete delineation of a four-stage diagnostic cascade: “chest pain → cardiac stent implantation → acute pancreatitis → pancreatic uncinate process carcinoma.” This real-world timeline serves as a stark warning against cognitive fixation and provides critical, practical insights for recognizing early warning signs and optimizing the diagnostic window in similarly complex clinical scenarios.

**Table 1:** Typical differences in pain caused by coronary heart disease, inferior myocardial infarction(IMI), acute pancreatitis and pancreatic cancer.

Feature	CHD	IMI	AP	PC
Pain quality	Pressure, squeezing, burning	Crushing, pressure-like, or may be absent	Persistent dull, sharp, or colicky	Dull ache or gnawing
Typical location	Retrosternal, precordial	Substernal, epigastric	Mid-epigastric	Mid-epigastric
Radiation	Left shoulder/arm	back, jaw, left shoulder/abdominal	Back, left shoulder/flank	Back, left shoulder/flank
Duration	Minutes to ~15 min (stable) or >20-30 min	Persistent until reperfusion (often >30 min)	Hours to days (continuous)	Early: Intermittent; Advanced: continuous; often nocturnal
Associated symptoms	Dyspnea, nausea, sweating, palpitations, fear of death, shock rarely	Diaphoresis, nausea, vomiting, syncope; hypotension and bradycardia	Nausea, vomiting, fever, bloating; shock in severe cases	Weight loss, poor appetite, new-onset or worsening diabetes, jaundice (head)
Physical exam	S3/S4 gallop, possible pericardial friction rub	Bradycardia, hypotension	Upper abdominal tenderness, guarding, rebound; hypoactive bowel sounds;	Late: palpable mass, jaundice, Courvoisier sign
Glucose metabolism	Stress hyperglycemia transient	Stress hyperglycemia transient	Grey-Turner or Cullen sign (severe)	New-onset diabetes or rapid worsening of pre-existing diabetes is a red flag;
Definitive test	Coronary angiography	Coronary angiography	Stress hyperglycemia transient CT ± EUS	EUS-FNA (tissue)

## Case presentation

A 65-year-old male presented with a 3-month history of intermittent, non-severe discomfort in the anterior chest region, occurring without obvious triggers and lasting several minutes per episode. He was admitted for evaluation after experiencing a frequent recurrence of this anterior chest discomfort accompanied by a pressing sensation over the precordium for two days. He reported no other symptoms such as abdominal pain, distension, shortness of breath, or hemoptysis.

His past medical history included essential hypertension for over 10 years, with a peak systolic blood pressure of 180 mmHg. His regimen consisted of Amlodipine Besylate 5mg daily, with adequate blood pressure control. He also had a prior episode of acute pancreatitis over a decade ago, with an unclear specific etiology, and a 5-year history of hyperlipidemia for which he had self-discontinued medication. Notably, the patient reported a significant alcohol and tobacco use history spanning more than 30 years.

On physical examination, the patient was afebrile and not in respiratory distress, with normal blood pressure readings. No bleeding spots or ecchymosis were found on the chest and abdomen. Cardiac auscultation revealed a slightly elevated heart rate with a regular rhythm and no abnormal sounds. Lung auscultation was clear bilaterally. Abdominal examination: soft, non−tender, no guarding or rebound tenderness; bowel sounds were normal in frequency and quality. No costovertebral angle tenderness was elicited.

The laboratory findings and their evolution process of multiple hospitalizations after onset of the disease can be seen in **Table 2**.

**Table 2:** The laboratory findings and their evolution process.

Laboratory findings;()	June 6th	July 12th	July 25th	August 8th	October 29th	Unit
WBC(4~10)	7.8	8	9.2	5.8		*10^9^/L
Hb(130~175)	149	131	130	119		g/L
PT(12~15)	12.9	12.8	13.4	13.9		Sec
D-2polymer (0~0.5)	0.37	0.38	0.5	0.4		mg/LFEU
hs-CRP(0~5)	3.5	2.05	9.62	2.28		mg/L
ALT(4~48)	13	32.5	46	35		U/L
AST(4~42)	19.1	28.8	27.7	29.3		U/L
TBil(5.1~20.5)	12.9	5.9	12	13.2		μmol/L
CA19-9(0~37)	**92.69**		**607.5**	**260.51**	**61.9**	U/mL
Amylase(25~125)	99	96	**515.5**	**179**	94	U/L
Serum lipase			**2328.2**			U/L
CK(22~269)	106.5	161.6	135.8	117.3		U/L
CK-MB(5~25)	10.8	15	18.7	13.2		U/L
hs-cTnl(<0.026)	0.002	0.002	0.007			μg/L
BNP(<100)	10.42	16	<5			pg/ml

Bold Values, significant abnormal values

### Timeline

**Figure 1:**
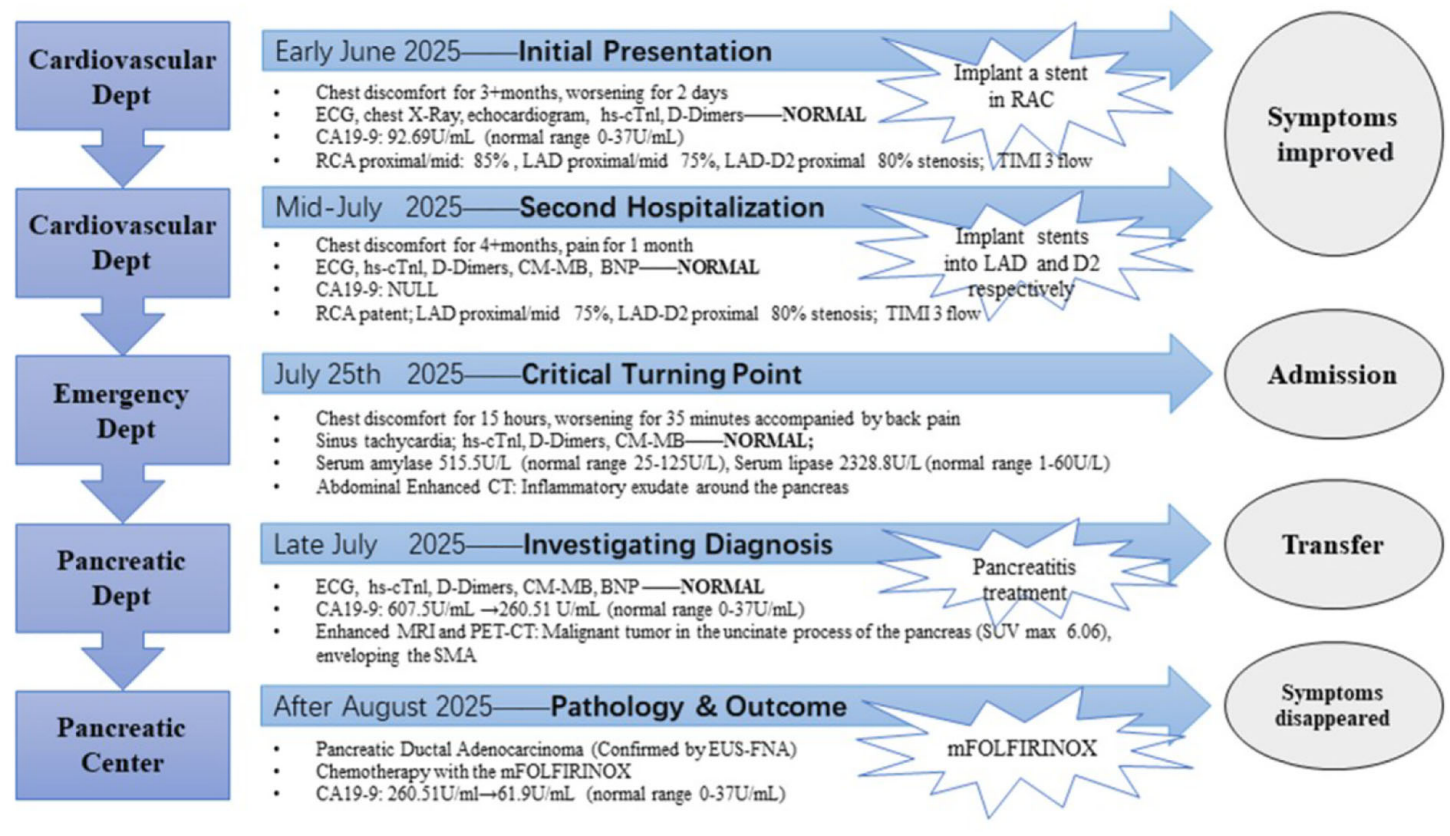
Diagnosis and treatment timeline.

The patient’s diagnosis and treatment course is illustrated in **Figure 1**.

### Diagnostic assessment

#### Initial assessment and therapeutic intervention

**Figure 2:**
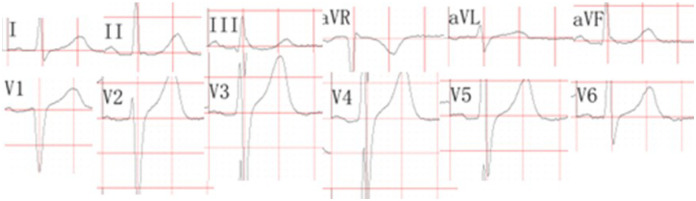
Resting ECG: the patient’s ECG was recorded at the moment that the patient complained of chest pain.

**Figure 3:**
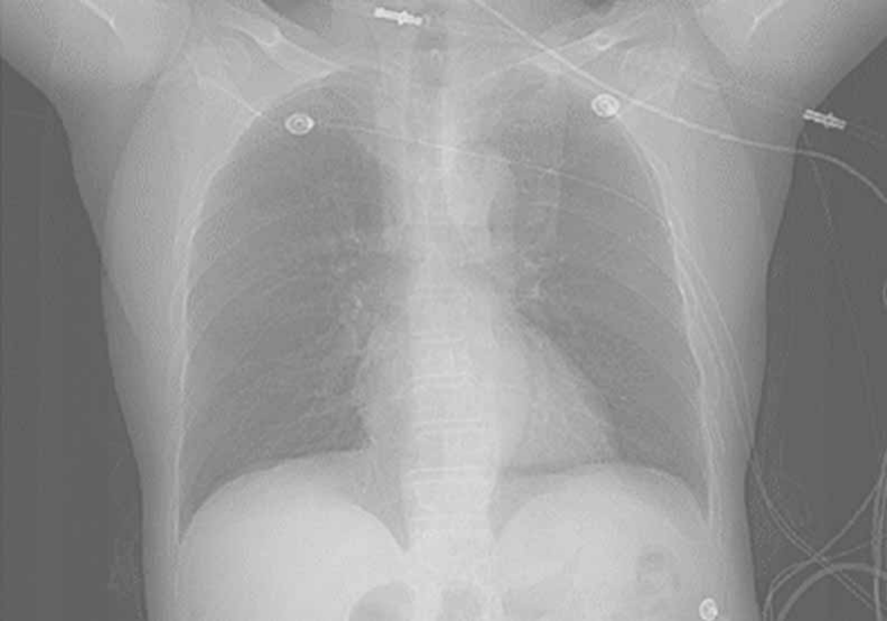
Chest X-ray shows a slightly rough shadow at the hilum of the lung.

**Figure 4:**
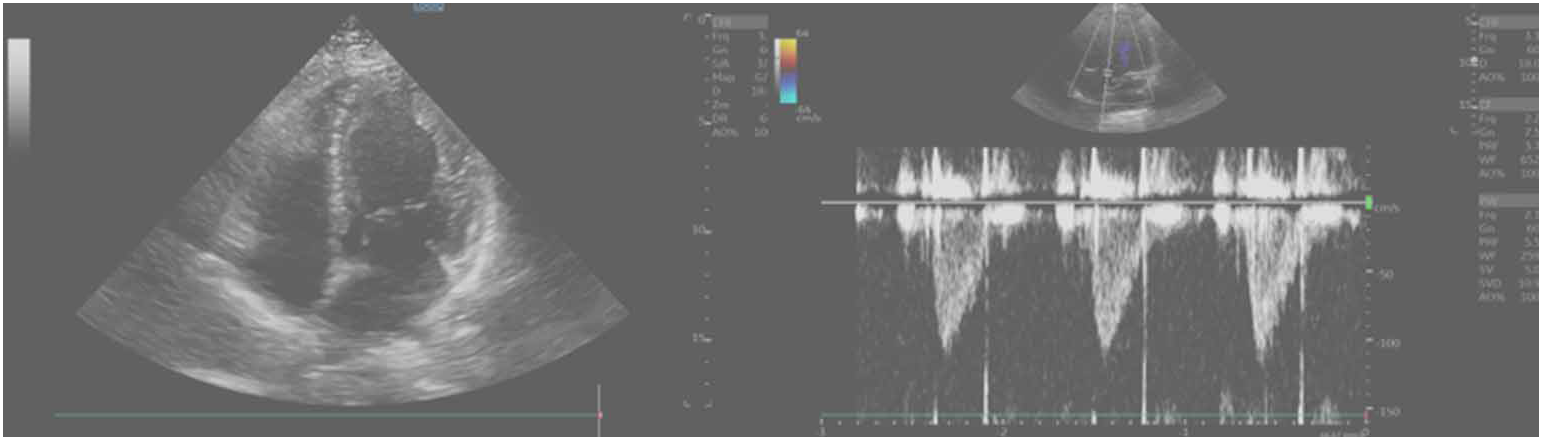
The patient’s echocardiogram upon initial admission.

**Figure 5:**
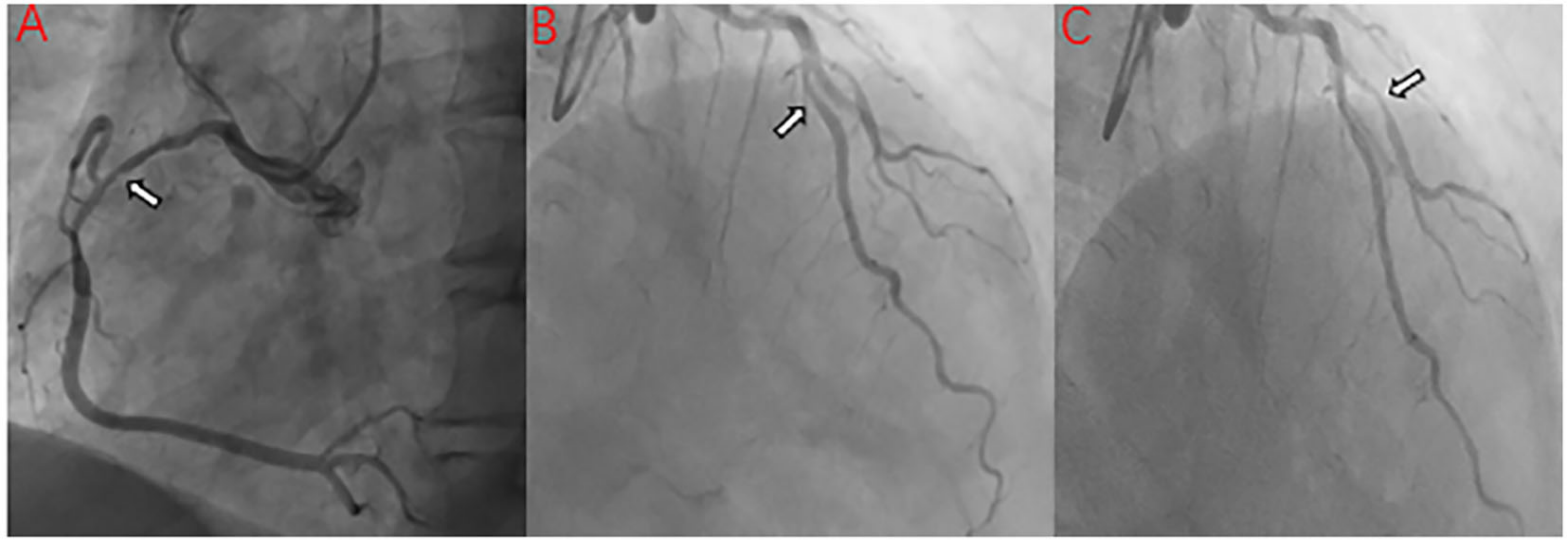
**(A)** 85% stenosis in the middle segment of the RAC; **(B)** The LAD has a stenosis of 75% in the middle segment; **(C)** About 80% stenosis at the proximal end of D2. TIMI 3 flow all.

The patient was initially admitted to the cardiology department with a presumptive diagnosis of CAD. However, apart from CA19-9, no significant abnormalities were found in the patient’s laboratory findings, electrocardiogram, chest X-ray, cardiac 2D and Doppler ultrasound (**Figures 2**–**4**). Based on the patient’s atypical symptoms, history of very high-risk hypertension, and significant smoking and alcohol use, the attending physician strongly suspected CAD. Following discussion with the family, a coronary angiography was performed directly. It revealed: a long lesion in the mid segment of the right coronary artery (RCA) with a maximum stenosis of 85%; no significant stenosis in the left main coronary artery; a long lesion in the proximal-to-mid segment of the left anterior descending artery (LAD) with up to 75% stenosis; approximately 80% stenosis at the origin of the second diagonal branch (D2); and 30% stenosis in the mid segment of the left circumflex artery. Thrombolysis in Myocardial Infarction (TIMI) flow grade 3 was maintained in all vessels (**Figure 5**). The interventional cardiologist decided on a staged procedure, initially deploying one drug-eluting stent in the RCA.

Approximately one month post-procedure, the patient was readmitted to the cardiology department due to persistent chest discomfort. Routine pre-operative examination prior to the second intervention showed no significant abnormalities; However, CA19–9 was not rechecked, and no abdominal imaging was performed. A repeat coronary angiography confirmed the RCA stent was patent without in-stent restenosis. Subsequently, a drug-eluting stent was placed in the mid LAD and another in the D2 branch during this second procedure. The patient was discharged uneventfully thereafter.

#### Diagnostic turn and discovery of acute pancreatitis

**Figure 6:**
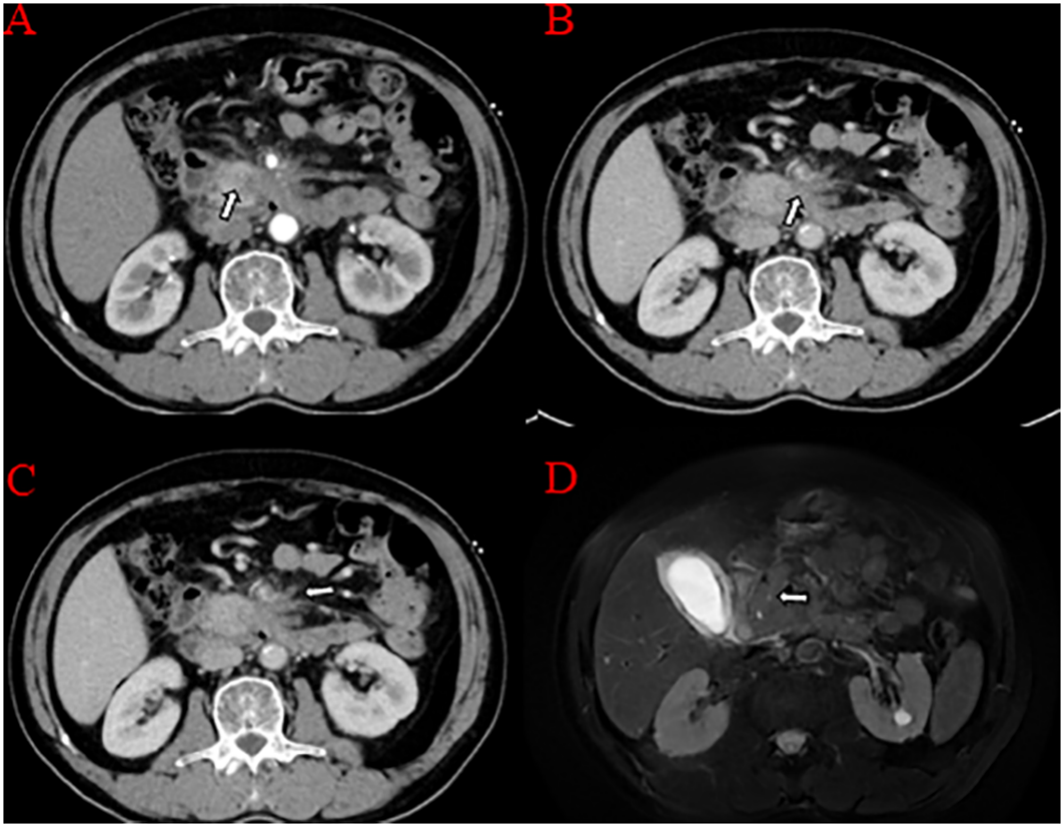
**(A)** Shows definite hypoenhancement in the arterial phase; **(B)**Tendency to invade surrounding fat, nerves, and vessels; **(C)** Inflammatory stranding and fascial thickening; **(D)** The tumor causes upstream pancreatic duct dilation with a “rat-tail” configuration or abrupt cutoff.

However, approximately two weeks after the second stent placement, the patient presented to the emergency department with recurrent chest pain accompanied by radiation to the back and lumbar region. This episode likely marked a pivotal diagnostic turning point. Emergency laboratory examination revealed a serum amylase level of 515.5 U/L and a markedly elevated lipase level of 2328.8 U/L. Contrast-enhanced computed tomography (CT) scan of the entire abdomen demonstrated inflammatory exudation localized to the uncinate process of the pancreas(**Figure 6C**). Following admission, further laboratory tests revealed a triglyceride level of 0.5 mmol/L and an immunoglobulin IgG4 level of 1.89 g/L. Most strikingly, the CA19–9 level had surged from the previous 92.69 U/mL to 607 U/mL. This disproportionate, several-fold increase in the tumor marker constituted a strong warning signal for an occult malignancy, especially in patients with acute pancreatitis of unknown cause (12).

**Figure 7:**
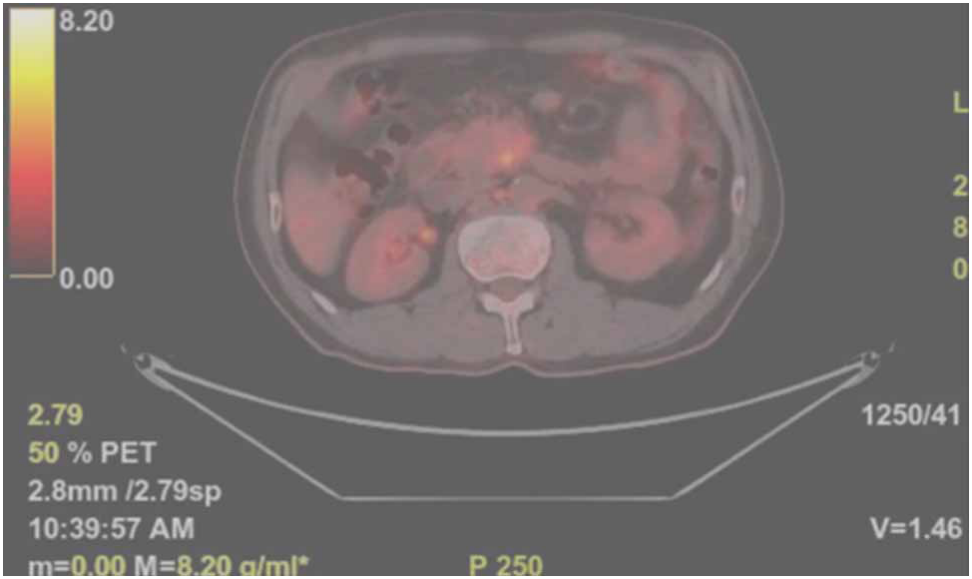
Typical PET-CT schematic diagram of the patient.

Doppler ultrasonography of the upper abdomen revealed heterogeneous pancreatic echogenicity and dilatation of the main pancreatic duct, up to 0.31 cm at its widest point. Although CT did not clearly identify a PC, it showed heterogeneous attenuation in the uncinate process with an indistinct border from the superior mesenteric artery (SMA)(**Figure 6A**). To minimize diagnostic interference from acute pancreatitis, the patient was managed with fasting, analgesia, digestive secretion suppression, and parenteral nutrition, leading to the resolution of the acute pancreatitis. Considering that CA19–9 remains high at 260.51 U/mL after retesting, we proceeded with pancreatic contrast-enhanced MRI and PET-CT scan to clarify the diagnosis. The findings aligned with our suspicion: an FDG-avid uncinate process lesion (SUVmax 6.06) encasing the SMA (**Figure 7**), with mild FDG-uptake lymphadenopathy (SUVmax 5.03) in the porta hepatis/pancreatic head and retroperitoneum.

#### Follow-up and outcome

The patient was transferred to a provincial pancreatic disease center, where endoscopic ultrasound-guided fine-needle aspiration (EUS-FNA) provided cytopathological evidence confirming the diagnosis of pancreatic ductal adenocarcinoma (cT4N1M0 stage III, AJCC 8th edition). Following four cycles of neoadjuvant chemotherapy with the mFOLFIRINOX regimen, a follow-up test showed a reduction of the CA19–9 level to 61.90 U/mL, and the patient’s chest pain had resolved.

## Discussion

The intricate diagnostic odyssey presented in this case is not incidental but rather the result of the interplay between systematic cognitive biases and the insidious nature of a specific disease. It compels clinicians to re-examine two pivotal questions: First, in the era of coronary intervention, has our technical confidence inadvertently narrowed the differential diagnostic horizon for chest pain, particularly in patients with ancillary laboratory abnormalities such as borderline elevation of tumor markers? Second, should the clinical presentation of pancreatic uncinate process carcinoma—with its dual masquerade of “chest pain” and “pancreatitis”—be recognized as a distinct syndrome warranting heightened vigilance? The following discussion will delve into an analysis of the diagnostic missteps and challenges in this case, aiming to extract universal lessons for clinical practice.

### Roots of misdiagnosis: anchoring bias and the limits of differential diagnosis in clinical decision-making

The initial evaluation in this case exemplifies a classic “anchoring bias.” When the patient presented with chest pain and coronary angiography confirmed multivessel coronary artery disease, the clinical reasoning became firmly anchored to the diagnosis of CAD (13). Consequently, the initially detected elevated CA19–9 level—a crucial abnormality—was discounted as incidental “noise” and failed to prompt an urgent investigation of abdominal viscera (14). However, the presence of confirmed coronary lesions should not preclude the consideration of comorbid conditions or other life-threatening pathologies as potential contributors to the clinical presentation (15). Therefore, early and proactive consideration of a broad differential diagnosis is essential to dislodge the cognitive anchor.

### The dual role of CA19-9: from an overlooked early warning signal to a pivotal monitoring marker

CA19–9 played a pivotal role in this case, its dynamic trajectory perfectly delineated the disease course from occult onset, through acute manifestation, to therapeutic response. While CA19–9 can be elevated in benign conditions such as biliary inflammation or pancreatitis, its persistent elevation or a disproportionate surge inconsistent with the clinical severity of inflammation serves as a strong signal indicative of an underlying malignancy. A practical differential pathway begins by excluding common benign causes through repeat testing and clinical correlation. Mild transient elevations often resolve with treatment of the benign condition. In contrast, a marked or persistently rising CA19–9 without a clear benign explanation—especially when accompanied by red flags such as new−onset diabetes, weight loss, or recurrent idiopathic acute pancreatitis—should prompt further imaging evaluation to rule out an occult malignancy (16, 17). This case imparts a critical lesson: even for patients presenting with chest pain, CA19–9 should be considered a crucial screening biomarker, and any abnormality warrants a definitive explanation. Furthermore, for acute pancreatitis with short-term recurrence and no obvious inducement, pancreatic tumor screening must be initiated, as approximately 10–20% of such cases are associated with an occult PC (18, 19). CA19–9 serves not only as a diagnostic clue but also as a sensitive tool for assessing therapeutic response and monitoring disease activity (20).

### The peculiarities of uncinate process pancreatic carcinoma: how anatomical location dictates clinical presentation

The clinical presentation of pancreatic uncinate carcinoma is highly deceptive. The uncinate process is situated deep in the retroperitoneum, in close proximity to the celiac plexus and the superior mesenteric vessels. Tumors in this location can infiltrate the neural plexus early, producing atypical radicular pain to the chest and back, which closely mimics cardiac angina or pleuritic pain (21). Concurrently, the tumor can obstruct the pancreatic duct, leading to obstructive pancreatitis. However, it does not directly impinge upon the common bile duct, early-onset jaundice is frequently absent (22). Acute pancreatitis attack caused by a mass in the pancreatic uncinate process, similar to the Roa Esparza case report (23).The combination of “neuropathic chest pain” and “obstructive pancreatitis” constitutes an atypical early syndrome of uncinate process carcinoma. The diagnostic odyssey in this case underscores the necessity for heightened clinical vigilance and precise selection of imaging modalities when evaluating tumors at this specific anatomical site.

### Imaging differentiation between uncinate process pancreatic carcinoma and mass-forming pancreatitis: diagnostic traps to be vigilant

For such cases presenting as acute pancreatitis, clinicians must acknowledge that radiographic “inflammation” may conceal a tumor and remain vigilant to atypical imaging signs. Especially in the differential diagnosis between mass-forming pancreatitis (MFP) and pancreatic ductal adenocarcinoma (PDAC), this challenge is exacerbated when the lesion is located in the uncinate process. They can all present as focal enlargement with abnormal density or signal intensity on imaging. The desmoplastic reaction surrounding PDAC often yields a hypovascular, ill-defined mass, whereas MFP, though capable of mimic these features, typically exhibits less aggressive vascular involvement. In pancreatic ductal adenocarcinoma. The subtle distinctions in their imaging features are key to differentiation, as detailed in **Table 3** (24, 25). When the nature of the lesion remains equivocal based on CT/MRI characteristics, EUS-FNA has become the definitive diagnostic step (12). EUS not only provides higher-resolution images for scrutinizing the internal architecture of the mass but also, through its guided tissue sampling, delivers conclusive pathological evidence, thereby closing the diagnostic loop and guiding correct clinical management (26).

**Table 3:** Key imaging differentiation points between MFP and PDAC in the uncinate process (24, 25).

Identifying dimensions	Mass-forming AP	PDAC (uncinate process)
Dynamic enhancement pattern	“Slow-in and slow-out” progressive enhancement	Hypovascular enhancement (**Figure 6A**)
Pancreatic duct alteration	“Duct-penetrating sign”	“Duct cutoff sign” “rat-tail” configuration “double-duct sign”
Peripheral invasion/reaction	inflammatory stranding and fascial thickening	infiltrative growth and encasement
Functional imaging (DWI-MRI)	apparent diffusion coefficient (ADC) value is typically mildly reduced or not significantly restricted	ADC value is significantly reduced
Evolution on follow-up	significantly decrease in size or resolve following anti-inflammatory treatment	the mass persists or progressively enlarges

## Conclusion

This case report, through an intricate diagnostic trajectory, underscores the paramount importance of maintaining a broad diagnostic perspective in the evaluation of chest pain, remaining vigilant for diseases with atypical presentations, conducting a systematic reassessment when symptoms recur after interventional procedures—particularly by looking beyond a cardiac-centric framework—and thoroughly investigating the underlying etiology of acute pancreatitis, especially when no common triggers are identified, with malignancy being a critical consideration. Even though recurrent chest pain in patients may be solely caused by coronary heart disease—it is our hope that documenting this four-stage diagnostic cascade will sharpen clinical vigilance against such cognitive pitfalls, optimize diagnostic pathways, and help prevent similar adverse outcomes.

## Data Availability

The original contributions presented in the study are included in the article/supplementary material. Further inquiries can be directed to the corresponding author.
